# The effect of social isolation on the cognitive ability of the oldest old in Chinese nursing homes in post-COVID-19: a moderated chain mediation model

**DOI:** 10.3389/fpsyg.2024.1421729

**Published:** 2024-09-02

**Authors:** Yi Fang, Linlin Ma, Huixian Chen, Shuya Cai, Wen Jiang, Fen Luo, Jialu Wang, Enjie Zheng, Chuncong Zhou, Lijuan Zhu, Qiaoqiao Guo, Zhiqin Yin

**Affiliations:** ^1^School of Nursing, Wenzhou Medical University, Wenzhou, China; ^2^The First Affiliated Hospital of Wenzhou Medical University, Wenzhou, China; ^3^Nursing Department, Naval Hospital of Eastern Theater, Zhoushan, China; ^4^School of Renji, Wenzhou Medical University, Wenzhou, China

**Keywords:** COVID-19, nursing home, oldest old, social isolation, cognitive ability, basic activities of daily living, depression, subjective socioeconomic status

## Abstract

**Background:**

Both pre-or post-COVID-19, older adults residing in nursing homes are at significant risk for social isolation, which is negatively associated with cognitive ability. Currently, the elderly aged 80 years and older are the fastest-growing age group globally. The extent of social isolation within this group post-COVID-19 and its impact on cognitive abilities remain inadequately explored.

**Objective:**

This research aimed to evaluate the prevalence of social isolation among the oldest old in Chinese nursing homes post-COVID-19 and to investigate the mediating and moderating roles of basic activities of daily living (BADL), depression, and subjective socioeconomic status in the relationship between social isolation and cognitive ability.

**Methods:**

This cross-sectional study included 453 participants aged 80 years and older from 11 nursing homes in Ningbo, Zhejiang Province, China. Social isolation was assessed using the Lubben Social Network Scale-6 (LSNS-6), cognitive ability using the Mini-Mental State Examination (MMSE), BADL using the Barthel Index (BI), and depression using the Patient Health Questionnaire-9 items (PHQ-9). Mediation and moderation effects were statistically analyzed using SPSS 23.0 and PROCESS 3.5.

**Results:**

The mean age of the study sample was 87.1 ± 3.8 years, among whom 60.3% (*n* = 273) were female, and 56.1% experienced social isolation, with 41.1% and 63.1% being isolated from family and friends, respectively. Social isolation indirectly affected cognitive ability through BADL and depression, respectively, and through the chain mediation effect of BADL and depression. Subjective socioeconomic status moderated the relationships between social isolation and BADL and between social isolation and depression. However, no moderating effect of subjective socioeconomic status was found between social isolation and cognitive ability.

**Conclusion:**

This study deepens our understanding of the current state of social isolation and its mechanisms of action in the oldest old post-COVID-19 and provides a new basis for future public health policy development and related research.

## 1 Introduction

Social isolation, defined as having few social relationships and infrequent social contact, has long posed a major challenge to global public health. Social isolation is highly, closely, and chronically associated with a variety of negative physical and mental health risks: hypertension, coronary heart disease, stroke, immune system compromise, anxiety, depression, cognitive deterioration, Alzheimer’s disease, and even untimely mortality (National Academies of Sciences Engineering and Medicine, 2020). Older adults face a heightened risk of social isolation due to reduced participation in social activities, a homogenization of social networks, transformations in traditional family structures, and functional limitations in physical activity (Sung et al., 2022). The COVID-19 pandemic further intensified social isolation, posing long-term and profound effects on the health and well-being of elderly people worldwide.

COVID-19 has been a calamity for nursing homes worldwide. To slow the spread of the virus, isolation measures seem to be a uniform practice globally (Ouslander and Grabowski, 2020). Various policies restricting visitor access and limiting resident–staff interactions have been implemented across countries and regions. Family visits were once essential for older adults to maintain social connections, but lockdown policies have deprived older adults in nursing homes of this social support, and these otherwise normal services and procedures are no longer available (Wu, 2020). Even after the pandemic, it seems that nursing homes continue to grapple with the challenge of balancing safety with openness. Stringent visitor restrictions remain essential for protecting resident health (Barnett and Grabowski, 2020). Although these measures were implemented out of necessity and consideration for the health of the elderly, they have elicited public concern and backlash over their potential to exacerbate the social isolation of institutionalized older persons (Chu et al., 2020; Stall et al., 2020).

Indeed, prior research has established that older adults residing in institutional settings are particularly susceptible to social isolation (Barbosa Neves et al., 2019). The institutionalized environment subjectively weakens their ties with members of their social networks and the outside community (Yan et al., 2022). Objectively, as an entity organization separate from healthcare, nursing homes often face a lack of convenient avenues for mental healthcare professionals to interact with and provide care for the residents (Blazer, 2020). The COVID-19 outbreak has undoubtedly dealt a devastating blow to the already fragile social networks of institutionalized older people. Within just 3 months following the inception of the outbreak, the prevalence of social isolation markedly increased, surpassing pre-pandemic levels (Su et al., 2023). This trend not only suggests an escalating rate of social isolation among this demographic but also indicates their increasing susceptibility to a spectrum of physical and psychological adversities (Stall et al., 2020; Bethell et al., 2021).

People aged 80 years and over (the oldest old) are the fastest-growing population group globally (Yang and Jia, 2022). Coinciding with this demographic shift is an accelerated rate of functional decline among the elderly, commencing from the age of 80, a phase during which health risks become increasingly significant (Dombrowsky, 2023). On this basis, their social role attributes will further diminish, and their social interactions will further decrease (Luhmann and Hawkley, 2016). Compared to younger older adults, the oldest old may face more prevalent and severe social isolation; for example, those aged 80 years and over are 2.41 times more likely to experience social isolation than those aged 60 and above (Wen et al., 2023). However, the extent of social isolation among the oldest old in Chinese nursing homes in the post-epidemic era remains unknown.

The stress hypothesis posits that social isolation acts as a significant stressor, with stress stimulation adversely affecting hippocampal structures and precipitating cognitive decline (Wilson et al., 2003). This phenomenon became particularly pronounced during and after the COVID-19 pandemic, wherein the social isolation induced by the outbreak precipitated a marked decline in cognitive ability among older adults (Corbett et al., 2023; Prommas et al., 2023). Although previous research has shed light on the effects of social isolation on cognitive ability, the specific experiences of social isolation among the institutionalized oldest old individuals in the post-COVID-19 era and the intricate mechanisms influencing cognitive ability remain underexplored. Clarifying these linkages not only provides a scientific basis for mitigating the negative effects of social isolation, but also lays a solid foundation for developing and implementing targeted intervention strategies to improve the cognitive ability and quality of life of the elderly and reduce the cost of care.

Hence, this study aims to evaluate the specific incidence of social isolation among the oldest old residents in Chinese nursing homes during the post-COVID-19 era and develop a mediation and moderation model elucidating the relationship between social isolation and cognitive ability.

## 2 Literature review and research hypotheses

### 2.1 Social isolation and cognitive ability

Cognitive ability encompasses an individual’s capacity to process and engage across various domains, including attention, perceptual speed, memory, language understanding and expression, and executive functions (Park and Reuter-Lorenz, 2009). Cognitive health pertains to the preservation of these faculties in a good state, thereby enabling the effective execution of daily life and social interactions. Cognitive reserve theory posits that social integration fosters mental stimulation through intricate communication and interactions with others, thereby building cognitive reserve (Bennett et al., 2006). Thus, social isolation leads to insufficient cognitive reserve development (Evans et al., 2018). The detrimental impact of social isolation on cognitive ability has been substantiated across various studies. Specially, longitudinal aging studies with extensive sample sizes across Europe, Asia, and the Americas show that social isolation is closely associated with diminished cognitive ability among older adults (Griffin et al., 2020; Read et al., 2020; Yu et al., 2021). Enhancing the social networks of older adults has been shown to prevent dementia and Alzheimer’s disease and improve cognitive ability (Fratiglioni et al., 2004; Pitkala et al., 2011). Nevertheless, despite the abundant studies on the effects of social isolation on cognitive ability, evidence concerning its implications within nursing homes, particularly among the oldest old, remains scarce. By synthesizing the existing research, we can infer the relationship between social isolation and cognitive ability among the institutionalized oldest old, leading us to propose the following hypothesis:

*H1:* Social isolation negatively predicts cognitive ability among the oldest old living in nursing homes.

### 2.2 The mediating role of basic activities of daily living

The biopsychosocial model underscores the complex interplay among biological factors, psychological states, and social environments in determining health and disease (Engel, 1977), thus revealing the limitations of a unidimensional approach in fully explaining the multifaceted nature of health. Consequently, when investigating the impact of social isolation on cognitive ability, a comprehensive and integrative perspective must be taken to uncover the underlying mechanisms.

The conceptual model of social network impact health posits that social relationships are vital resources, fostering improved physical and mental health (Berkman et al., 2000). Studies have consistently demonstrated that expansive social networks are crucial for maintaining health, with individuals possessing wider social networks experiencing less physical decline (Ali et al., 2018). Basic activities of daily living (BADL) is a core indicator for assessing physical functioning in older adults (Edemekong et al., 2002). It includes the ability to eat, dress, bathe, control defecation, and walk, reflecting the individual’s independence and functional status in performing self-care and daily tasks (Katz et al., 1963). Previous evidence shows that social isolation is associated with lower levels of BADL in the past, present, and even future (Moreno-Tamayo et al., 2020). Older women with high social isolation show more pronounced BADL impairment (Guo et al., 2021a). The COVID-19 pandemic, characterized by enforced social isolation, has exacerbated functional limitations in older adults, leading to various health problems, including cognitive impairment (Fettes et al., 2021; Laura Frutos et al., 2023).

Most studies on cognitive impairment have focused on exploring the negative effects of cognitive impairment on BADL (Arora et al., 2023), potentially overlooking the critical role of physical functioning in maintaining cognitive ability and the impact of social attributes. From a use-it-or-lose-it perspective, diminishing BADL restricts activity and social interactions in older adults, thereby reducing cognitive stimulation and accelerating cognitive decline (Bielak and Gow, 2023). One meta-analysis found that individuals with decreasing BADL are at a higher risk of developing dementia than individuals with cognitive impairments who maintain their functional abilities (Jekel et al., 2015). Accordingly, the following hypothesis is proposed:

*H2:* BADL mediates the relationship between social isolation and cognitive ability.

### 2.3 The mediating role of depression

Mental state is an integral part of the biopsychosocial model. The social convoy model suggests that social networks can help individuals at different life stages, promoting their physical and mental health and escorting them to a healthy life (Antonucci et al., 2014). Good social network relationships, network structure, and participation in social activities are frequently associated with better mental health outcomes. Conversely, poor social network relationships breed negative emotions (Cornwell and Laumann, 2015).

The World Health Organization has highlighted that the COVID-19 pandemic has disproportionately led to negative emotions among older adults by affecting multiple aspects of their ability to connect socially (World Health Organization, 2020). Depression is one of the most prevalent psychiatric conditions among the elderly (Qin et al., 2018). A large body of research suggests older adults who lack adequate social networks are at a higher risk of developing depression (Robb et al., 2020; Santini et al., 2020; Quach and Burr, 2021). Although depression is primarily characterized by negative affect, cognitive impairment is increasingly recognized as a common symptom in people with depression (Motter et al., 2016). Approximately two-thirds of older depressed patients experience cognitive impairment (Afridi et al., 2011). Longitudinal studies have consistently demonstrated that depression predicts reduced cognitive performance and further cognitive decline over time (Yang et al., 2020; Zhou et al., 2021; Huang et al., 2022). In older adults with depression, social isolation and decreased cognitive ability are significantly associated in cross-sectional and longitudinal studies (Evans et al., 2019; Guo et al., 2021b). Against this background, the following hypothesis is proposed:

*H3:* Depression mediates the relationship between social isolation and cognitive ability.

### 2.4 Chain mediation of BADL and depression

BADL and depression are key physical and psychological dimensions, respectively, among older adults, and both may be intensified by social isolation. Research has suggested that older adults with restricted BADL are less likely to engage in social activities and lack sufficient interpersonal communication, which may increase their risk of depression (Chao et al., 2024). Moreover, being unable to perform basic life activities independently can make older adults feel powerless and dependent, fostering distress and pessimism (Wang et al., 2023). Although previous research on the relationship between BADL and depression has yielded varying mechanisms of influence, cross-sectional and longitudinal studies consistently show a robust association between decreased BADL and depression (Tian et al., 2022; Yan et al., 2023; Zhou et al., 2024). Previous research suggests that decreased BADL is an important correlate of depression risk in older adults (Liu et al., 2023), and prevention of BADL disability may positively impact medical care for older adults with depression (Feng et al., 2021).

Despite these insights, no research has explicitly explored whether BADL and depression may act as chain mediators in the relationship between social isolation and cognitive ability. Therefore, considering previous research findings, the following hypothesis is proposed:

*H4:* Social isolation impacts cognitive ability through the chain-mediated effects of BADL and depression.

### 2.5 The moderating role of subjective socioeconomic status

While social isolation may impact cognitive ability through BADL and depression, this effect may vary by socioeconomic status. Recent literature recognizes socioeconomic status as a critical factor of social inequality and suggests that COVID-19 has further exacerbated inequalities in later life (Blundell et al., 2020; González Rodríguez et al., 2022; Si et al., 2023). The network theory of social capital posits that the strength and breadth of social ties, along with the availability of social resources, vary considerably across socioeconomic groups, with individuals of higher socioeconomic status tending to have wider networks of friends and family, stronger social ties, and more social resources, resulting in greater social support (Lin, 2019). These differences lead to differences in physical and mental health (Wang and Matz-Costa, 2019). Such as, patients with rich social support networks are able to perceive more social support, which leads to better adherence and healthier behaviors (Yang et al., 2024).

However, socioeconomic status is often not sufficiently considered in previous research on the impact of social isolation on health. The few studies that consider socioeconomic status have produced inconsistent findings. While some studies suggest stronger associations between social isolation and health indicators in groups with lower socioeconomic status (Lai et al., 2023), others indicate that social isolation may constitute a more serious health risk for more economically privileged populations (Luo et al., 2021). Still, previous research suggests that the health effects of social isolation differ significantly according to socioeconomic status. Furthermore, research on this issue in the post-COVID-19 era is lacking.

Most research has quantified socioeconomic status through objective measures such as income; however, perceived socioeconomic status can differ significantly among individuals with equivalent incomes. According to social comparison theory, an individual’s subjective sense of deprivation through comparison with others may be more detrimental to their physical and mental health than the objective reality of the situation (Wilkinson and Pickett, 2007). Recent studies support this, showing that subjective socioeconomic status better reflects an individual’s perceptions than does objective income (Tan et al., 2020). Therefore, this study used subjective socioeconomic status as an indicator to measure the economic conditions of the oldest old. The following hypotheses are proposed:

*H5:* Subjective socioeconomic status moderates the relationship between social isolation and BADL.

*H6:* Subjective socioeconomic status moderates the relationship between social isolation and depression.

*H7:* Subjective socioeconomic status moderates the relationship between social isolation and cognitive ability.

**Figure 1:**
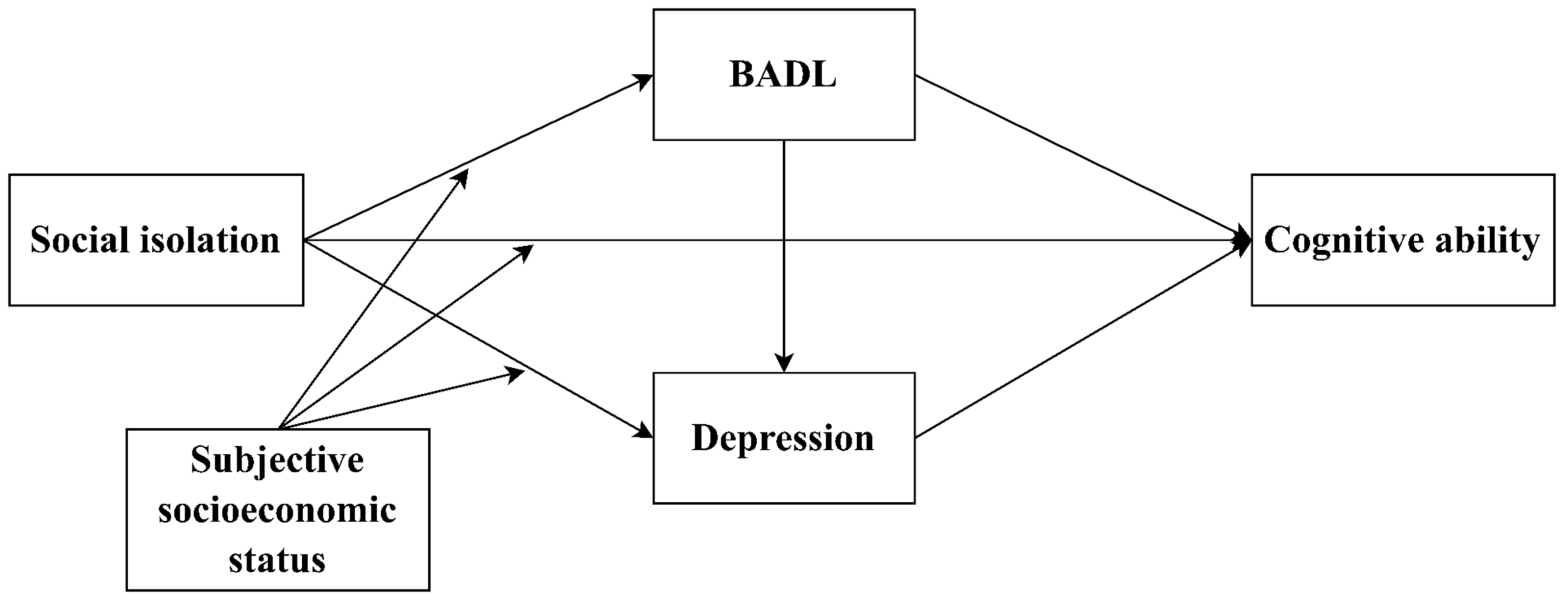
Hypothesized relationships of the research model. BADL, basic activities of daily living.

In summary, this study aims to examine the prevalence of social isolation and its pathways to cognitive ability among the oldest old in nursing homes in China during the post-COVID-19 era. This study will contribute to the existing research field in three ways. Firstly, previous research on this issue is scarce. Secondly, although previous studies have explored different pathways of the effects of social isolation on cognitive ability, validating the mediating role of depression somewhat, research considering the role of BADL and its effects on depression is quite limited. Therefore, this study is the first attempt to validate a chain mediation model of the effects of social isolation on cognitive ability among the oldest old in nursing homes. Finally, the present study explores the role of different subjective socioeconomic statuses in this chain mediation model to further expand the theoretical and empirical foundations of research on how the relationship between social isolation and health factors differs by subjective socioeconomic status among the oldest old. Based on relevant theories and research, we constructed the hypothetical model shown in Figure 1.

## 3 Methods

### 3.1 Participants and procedures

Between July 2023 and January 2024, this study conducted a cross-sectional survey using convenience sampling in 11 nursing homes in Ningbo, Zhejiang Province, China. These sampled nursing homes represent 5.5% of the total nursing homes in Ningbo (201 nursing homes were registered in Ningbo as of June 2023, according to the People’s Government of Zhejiang Province). The survey respondents were the oldest old, aged ≥80 years, who had been in the institutions for ≥6 months. We excluded participants with: (1) severe language or hearing impairments that prevented them from understanding or responding to questions; (2) severe visual impairments that could compromise their safety during physical tests or assessments; (3) medically documented severe cognitive impairments (e.g., advanced Alzheimer’s, severe vascular dementia); (4) diagnosed severe psychiatric disorders (e.g., schizophrenia, severe anorexia nervosa, obsessive-compulsive disorder); (5) current acute comorbidities (e.g., myocardial infarction); and (6) extreme frailty or end-of-life status. Medical records were obtained from participants’ institutions with physician assistance, ensuring ethical compliance and data accuracy.

Professionally trained investigators read the informed consent form to participants, detailing the purpose, significance, anonymity, and confidentiality of the study and assuring them of their right to withdraw from the study at any time. These investigators obtained informed written consent from all participants, either personally or by authorization, and data were collected via face-to-face interviews by investigators to ensure that participants fully understood the meaning of survey questions before investigators filled in the responses.

In our study, We employed a dual strategy to determine the required sample size. Using G*Power 3.1, we estimated a minimum of 166 valid samples needed for our multiple linear regression model (effect size = 0.15, *α* = 0.05, power = 0.95,and including 9 predictors). To safeguard against potential sample attrition or invalidity, we increased this number by 20%, aiming for at least 199 participants. Additionally, based on our methodological framework and literature on multi-step structural equation model, we targeted a sample size between 221 and 1,000 (Sim et al., 2022). Ultimately, 485 questionnaires were distributed with a response rate of 93.4% (*n* = 453). Among the 453 participants in the final sample, 273 were female (60.3%), and the mean age was 87.13 years (SD = 3.84), ranging from 80 to 100. The study followed the Helsinki Declaration of Ethical Principles and was approved in its entirety by the Ethics Review Committee of Wenzhou Medical University (Ethics approval number: 2023-005). Basic participant information is shown in Table 1.

**Table 1:** Distribution and differences of social isolation by sociodemographic and health characteristics.

Variables		*N* (%)	SI		
			*M* (SD)	*F/t*	*p*
Gender	Male	180 (39.7)	11.41 (5.19)	3.939	<0.001
	Female	273 (60.3)	9.36 (5.72)		
Age group	80–84 years	118 (26.0)	14.16 (4.90)	83.054	<0.001
	85–89 years	207 (45.7)	10.31 (4.72)		
	≥90 years	128 (28.3)	6.27 (4.84)		
Marriage	Married	140 (30.9)	13.62 (5.05)	9.605	<0.001
	Unmarried	313 (69.1)	8.63 (5.14)		
Education	High school or above	56 (12.4)	15.14 (3.54)	113.396	<0.001
	Junior high school	98 (21.6)	13.74 (4.37)		
	Primary	133 (29.4)	11.08 (4.40)		
	Below primary	166 (36.6)	5.66 (4.23)		
Duration of residence	< 1 year	52 (11.5)	11.79 (5.66)	9.205	<0.001
	1–2 years	75 (16.6)	10.47 (5.37)		
	3–5 years	163 (36.0)	10.99 (5.05)		
	6–10 years	97 (21.4)	10.10 (5.81)		
	> 10 years	66 (14.6)	6.64 (5.53)		
BADL	No disability	82 (18.1)	16.65 (3.00)	259.033	<0.001
	Mild disability	245 (54.1)	11.22 (3.86)		
	Moderate disability	41 (9.0)	6.32 (3.42)		
	Severe disability	85 (18.8)	2.75 (2.08)		
SES	Low	121 (26.7)	4.12 (3.07)	−21.914	<0.001
	High	332 (73.3)	12.38 (4.61)		

*N* = 453. SI, social isolation; BADL, basic activities of daily living; SES, subjective socioeconomic status.

### 3.2 Measurements

#### 3.2.1 Social isolation

Social isolation (SI) was measured using the Lubben Social Network Scale (LSNS-6) (Lubben et al., 2006). This scale has been widely used to study the elderly Chinese population and has shown good reliability and validity (Chang et al., 2018; Sun et al., 2022). The six entries of the scale were further divided into two dimensions—family network and friend network—measuring the number of social contacts from family and friends and the level of perceived social support. On the family network dimension, participants were asked, (a) “How many family members have you seen in the last month?” (b) “How many family members were able to seek help for you?” (c) “How many family members can you talk to about personal matters?” (For friend networks, “family” was substituted for “friends,” and these three items were repeated). A 6-point Likert scale was used to rate each item, and participants had to choose from 0 to 6 points, which were summed to obtain a total score. Thus, the total score of LSNS-6 ranges from 0 to 30, with higher scores representing larger social networks and less social isolation. A total score of <12 points indicates being isolated. Cut-off scores for the two subscales of family and friends were validated at score 6 to best discriminate between isolated and non-isolated people; respondents with a score of less than 6 points are defined as socially isolated with regard to the respective context (Lubben et al., 2006). In this study, the Cronbach’s α for family and friend networks were 0.850 and 0.885, respectively.

#### 3.2.2 Cognitive ability

Cognitive ability (CA) was assessed using the Mini-Mental State Examination (MMSE) developed by Folstein et al. (1975). The MMSE is the most commonly used screening tool for cognitive impairment worldwide (Shulman et al., 2006), and has been validated in Chinese older adults (Zhang et al., 2006; Lv et al., 2019). The scale evaluates five areas of ability, including orientation, memory, calculation, recall, and language ability, with a total of 30 questions, with each question scored 1 point for a correct answer and a total score of 0 to 30, with higher scores representing better cognitive ability. In the present study, Cronbach’s α was 0.924.

#### 3.2.3 Basic activities of daily living

The Barthel Index (BI) has been used to assess BADL (Mahoney and Barthel, 1965). The scale has been reported to have high reliability and validity in the Chinese elderly population (Feng et al., 2013; Zhang et al., 2022). The scale consists of 10 items, including feeding, bathing, personal hygiene, dressing, toileting, controlling bowel, controlling urination, transferring bed and chair, walking on level ground, and going up and down stairs, with each item being assigned a score of 0, 5, 10, or 15, ranging from complete dependence to self-care. Total scores range from 0 to 100, with higher scores indicating greater independence. No disability scores 100 points, mild disability 61–99 points, moderate disability 41–60 points, and severe disability 40 points and below. In this study, Cronbach’s α was 0.947.

#### 3.2.4 Depression

The Patient Health Questionnaire-9 items (PHQ-9) was used to assess the depression. The scale consists of 9 items, and a 4-point Likert scale was used to measure the psychological/physical condition from the past 2 weeks and the frequency. The PHQ-9 score can range from 0 to 27, with each of the 9 items being scored from 0 (not at all) to 3 (nearly every day) and higher scores indicating a higher level of depression (Kroenke et al., 2001). The scale has been validated for Chinese older adults (Gao et al., 2023). In this study, Cronbach’s α was 0.943.

#### 3.2.5 Subjective socioeconomic status

Subjective socioeconomic status (SES) refers to an individual perception of their position in the socioeconomic structure or social hierarchy (Tan et al., 2020). We measured subjective socioeconomic status by asking, “How do you perceive your economic status compared to everyone you know?” This method of questioning aims to simplify the question and effectively capture the intuitive feelings of the oldest old about their economic status. Thus, participants responded either “high” or “low” to express their subjective assessment of their socioeconomic status.

### 3.3 Control variables

According to previous research, the sociodemographic variables of gender (Umberson et al., 2022), age (Silberzan et al., 2022), marriage (Haggerty et al., 2022), education (Fernández-Carro and Gumà Lao, 2022), and duration of residence (Chamberlain et al., 2020) were identified as possible confounders of cognitive ability.

Sex was categorized as male or female; age was categorized as 80–84, 85–89, and ≥ 90 years; marriage was categorized as married or unmarried; education was categorized as below primary, primary, junior high school, and high school or above; and duration of residence was categorized as <1 year, 1–2 years, 3–5 years, 6–10 years, and > 10 years. Participants’ ages were confirmed based on the date of birth on their People’s Republic of China Resident Identity Cards. The duration of residence was obtained from medical record documents kept by the institution.

### 3.4 Statistical analysis

All data analyses were conducted using IBM SPSS for Windows, version 23 (IBM Corp., Armonk, NY, United States). In descriptive statistics, frequencies (percentages) and means (standard deviations) were used for categorical and continuous variables, respectively. *T*-tests and one-way analyses of variance (ANOVA) were used to explore differences between sociodemographic and health variables and social isolation. In addition, Pearson correlation analyses were used for cognitive ability, social isolation, BADL, and depression to assess the degree of correlation between these variables, and a multicollinearity test was used to determine whether there was an issue of multicollinearity between the variables.

Models 6 and 85 of the SPSS macro PROCESS, version 3.5, were used to analyze mediating and moderating effects (Hayes, 2017). Total scores for social isolation (independent variable), cognitive ability (dependent variable), BADL (mediator 1), and depression (mediator 2) were included in the models. A 95% confidence interval (CI) and 5,000 bootstraps were utilized for these analyses. If the 5,000-bootstrap 95% CI did not include 0, the indirect effect was statistically significant. In addition, if a statistically significant interaction term was evident, simple slope analyses (pick-a-point method) were conducted to demonstrate the moderating role. The bootstrap method was similarly used to assess the significance of indirect effects between high/low subjective socioeconomic status (moderator). In all mediation and moderation model analyses, covariates, including age, gender, marriage, education, and duration of residence, were controlled. A two-tailed test was used with a statistical significance level of *p* < 0.05 and a 95% CI.

## 4 Results

### 4.1 Test for common method bias

In this study, we utilized self-report scales to collect data. To ensure the robustness, reliability, and validity of our conclusions, we carefully evaluated the potential impact of Common Method Bias (CMB) on the research outcomes. Through the Harman’s single-factor test, we identified 8 independent factors with eigenvalues exceeding 1, and the variance explained by the first factor was 44.4%, which is below the widely accepted threshold of 50% (Podsakoff et al., 2003). This result indicates that the data in this study were not significantly influenced by common method bias.

### 4.2 Descriptive statistics

The percentage of participants with a total social isolation score < 12 points (being isolated) was 56.1% (*n* = 254), with the prevalence of isolation from family and friends being 41.1% (*n* = 186) and 63.1% (*n* = 285), respectively. Table 1 shows the distribution of and differences in social isolation by demographic and health variables. The results showed that there were statistically significant differences between social isolation and gender, age, marriage, education, duration of residence, BADL, and subjective socioeconomic status (*p* < 0.001).

### 4.3 Correlations analysis

The means, standard deviations, and correlation coefficients of the main variables are shown in Table 2. While cognitive ability, social isolation, and BADL were positively correlated (*p* < 0.01), depression was negatively correlated with cognitive ability, social isolation, and BADL (*p* < 0.01).

**Table 2:** Descriptive statistics and correlation matrix for the main variables.

Variables	M ± SD	1	2	3	4
1.CA	25.03 ± 5.67	–			
2.SI	10.17 ± 5.60	0.786^**^	–		
3.BADL	72.26 ± 28.55	0.808^**^	0.798^**^	–	
4.Depression	5.96 ± 7.67	−0.798^**^	−0.805^**^	−0.873^**^	–

^**^ *p* < 0.01. *N* = 453.CA, cognitive ability; SI, social isolation; BADL, basic activities of daily living.

### 4.4 Chain mediation effect analysis

Before performing the regression analysis, the multicollinearity test for the main variables found that the maximum value of variance inflation factor (VIF) was 4.861, which was <5, indicating no serious issues of multicollinearity among these variables.

The regression results of chain mediating effects are shown in Table 3. Both social isolation (*β* = 0.191, *p* < 0.001) and BADL (*β* = 0.040, *p* < 0.001) positively predicted cognitive ability, whereas depression negatively predicted cognitive ability (*β* = −0.225, *p* < 0.001). Furthermore, both social isolation (*β* = −0.421, *p* < 0.001) and BADL (*β* = −0.182, *p* < 0.001) negatively predicted depression, whereas social isolation positively predicted BADL (*β* = 3.264, *p* < 0.001).

**Table 3:** Regression results of chain mediating effects.

Variables	Model 1		Model 2		Model 3	
Outcome	BADL		Depression		CA	
Predictor	*β*	*t*	*β*	*t*	*β*	*t*
Gender	−3.112	−1.931	0.749	2.220^*^	−0.680	−2.395^*^
Age	−1.664	−6.019^***^	−0.291	−4.851^***^	−0.346	−6.736^***^
Education	−1.856	−1.910	0.283	1.393	−0.624	−3.661^***^
Marriage	5.040	2.722^**^	−0.052	−0.134	0.479	1.471
Duration of residence	−1.236	−1.642	0.284	1.805	0.228	1.728
Constant	189.720	7.760^***^	45.874	8.452^***^	53.063	10.845^***^
SI	3.264	15.639^***^	−0.421	−7.782^***^	0.191	3.964^***^
BADL			−0.182	−18.408^***^	0.040	3.689^***^
Depression					−0.225	−5.673^***^
*R*^2^		0.682		0.809		0.756
*F*		159.378^***^		269.802^***^		171.899^***^

^*^ *p* < 0.05,^**^ *p* < 0.01,^***^ *p* < 0.001. *N* = 453.BADL, basic activities of daily living; CA, cognitive ability; SI, social isolation.

The results of the chained mediation effects analysis are presented in Table 4. The mediating effect of BADL and depression was significant, with a total mediation effect value of 0.360. Specifically, the effect of social isolation on cognitive ability was influenced through three indirect pathways, all of which reached the level of significance. The indirect pathway one refers to SI → BADL→CA, with an indirect effect of 0.132, 95% CI = [0.045, 0.228], excluding 0, indicating a significant mediating effect of BADL. SI → Depression→CA was indirect pathway two with an indirect effect of 0.095, 95% CI = [0.046, 0.153], excluding 0, indicating a significant mediating effect of depression. The last indirect pathway was the chain mediation with BADL and depression (SI → BADL→Depression→CA), and the indirect effect was 0.133, 95% CI = [0.076, 0.191], also excluding 0, suggesting that BADL and depression played a significant role in the chain. The chain mediating effect accounted for 24.1% of the total, and the independent mediating effects of BADL and depression accounted for 23.9% and 17.2%, respectively. Thus, H1, H2, H3, and H4 were supported.

**Table 4:** BADL and depression in the chain mediation analysis.

Pathway	Effect	Bootstrap SE	Bootstrap 95% CI		Effect size
			Lower	Upper	
Ind1: SI → BADL→CA	0.132	0.047	0.045	0.228	23.9%
Ind2: SI → Depression→CA	0.095	0.027	0.046	0.153	17.2%
Ind3: SI → BADL→ Depression →CA	0.133	0.029	0.076	0.191	24.1%
Total Indirect Effect	0.360	0.039	0.284	0.441	65.2%
Direct Effect	0.191	0.049	0.096	0.286	34.6%
Total Effect	0.552	0.043	0.466	0.635	100.0%

*N* = 453.BADL, basic activities of daily living; CA, cognitive ability; SI, social isolation.

### 4.5 The moderated chain mediation analysis

**Figure 2:**
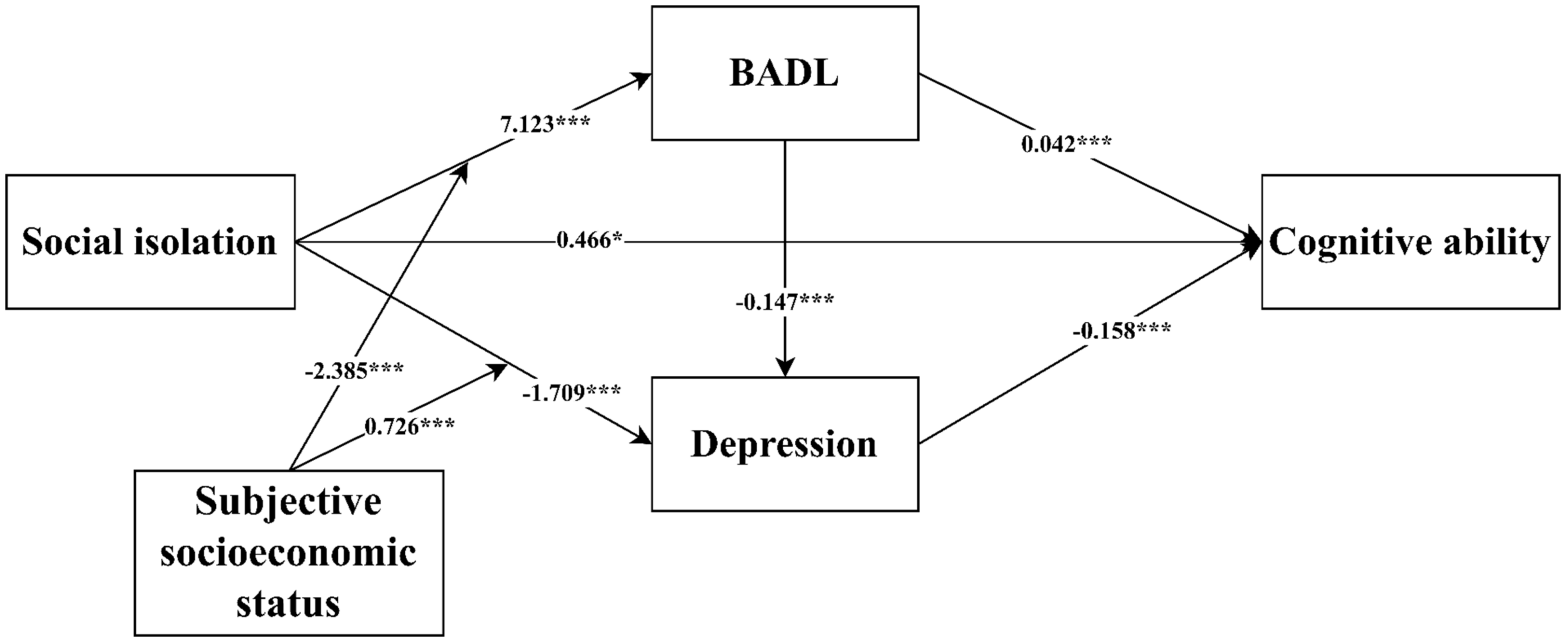
Path coefficients for the moderated mediation model. *p<0.05, ***p<0.001. *N* = 453. BADL, basic activities of daily living.

The regression results of the moderated chain mediation model are shown in Table 5. The significance of the coefficients of the chained mediator was not affected when including the moderator, whereas the interaction terms of social isolation and subjective socioeconomic status significantly affected BADL (*β* = −2.385, *t* = −4.725, *p* < 0.001) and depression (*β* = 0.726, *t* = 7.154, *p* < 0.001), which suggests that subjective socioeconomic status plays a moderating role in both of these; therefore, H5 and H6 were supported. However, the results showed that cognitive ability could not be predicted by the interaction term of social isolation and subjective socioeconomic status (*β* = −0.149, *t* = −1.492, *p* = 0.136); therefore, H7 was not supported. Figure 2 illustrates the final significant moderated mediated path coefficient diagram.

**Table 5:** Regression results of the moderated chain mediation model.

Variables	Model 1		Model 2		Model 3	
Outcome	BADL		Depression		CA	
Predictor	*β*	*t*	*β*	*t*	*β*	*t*
Gender	−2.887	−1.880	0.787	2.603^*^	−0.785	−2.766^**^
Age	−1.846	−7.033^***^	−0.178	−3.274^**^	−0.366	−7.145^***^
Education	−0.608	−0.634	−0.031	−0.163	−0.498	−2.838^**^
Marriage	3.942	2.248^*^	0.107	0.310	0.440	1.364
Duration of residence	0.020	0.027	−0.055	−0.383	0.311	2.329^*^
Constant	161.56	6.866^***^	47.873	9.862^***^	49.896	9.989^***^
SI	7.123	7.411^***^	−1.709	−8.552^***^	0.466	2.318^*^
BADL			−0.147	−15.796^***^	0.042	3.856^***^
Depression					−0.158	−3.559^***^
SES	25.764	7.424^***^	−7.828	−10.847^***^	2.330	3.080^**^
Interaction^#^	−2.385	−4.725^***^	0.726	7.154^***^	−0.149	−1.492
*R*^2^	0.718			0.850		0.762
*F*	141.429^***^		279.582^***^		141.822^***^	

^#^Interaction refers to the interaction term between SI and SES; ^*^ *p* < 0.05, ^**^ *p* < 0.01, ^***^ *p* < 0.001. *N* = 453.BADL, basic activities of daily living; CA, cognitive ability; SI, social isolation; SES, subjective socioeconomic status.

**Figure 3:**
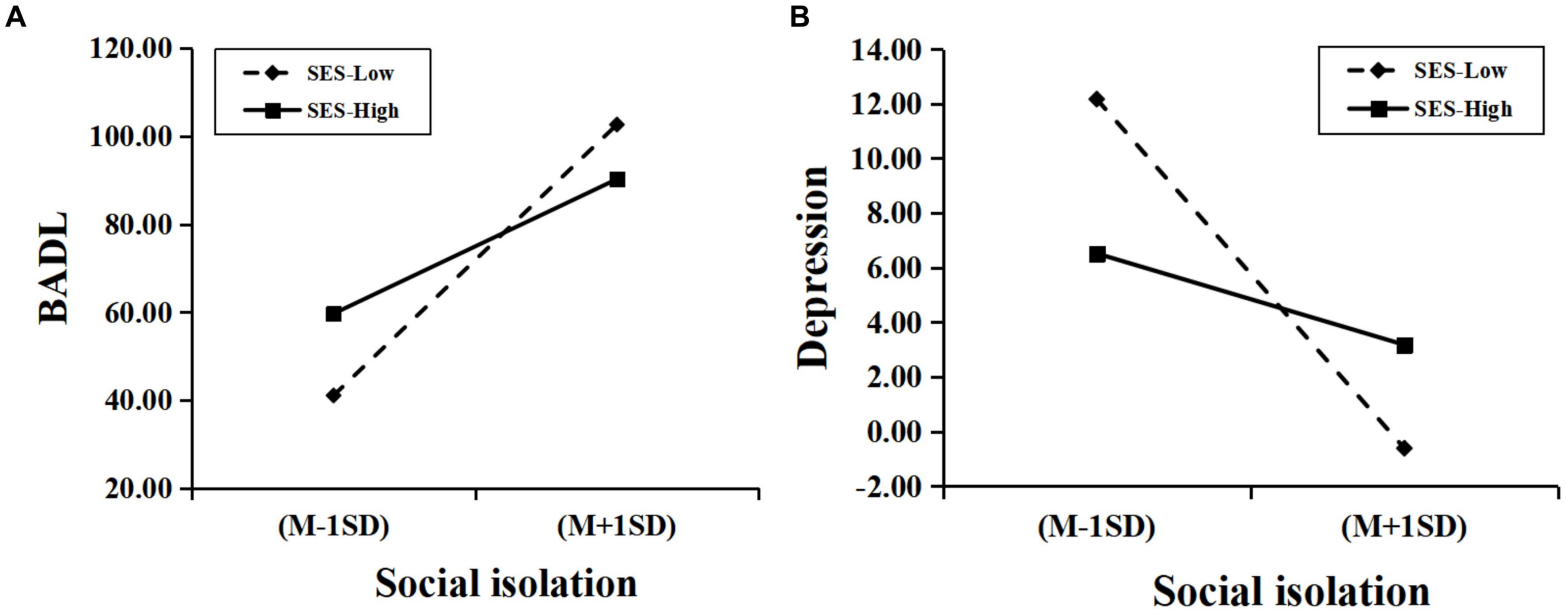
Moderating effects of subjective socioeconomic status between social isolation and BADL and depression. **(A)** the moderating effect of subjective socioeconomic status between social isolation and BADL; **(B)** the moderating effect of subjective socioeconomic status between social isolation and depression. *N* = 453. BADL, basic activities of daily living; SES, subjective socioeconomic status.

To further analyze how subjective socioeconomic status moderated social isolation with BADL and depression, the simple slope test was performed. As shown in Figure 3, social isolation was a significant positive predictor of BADL for participants with low subjective socioeconomic status (*β*_simple_ = 7.123, *t* = 7.479, *p* < 0.001). For those with higher levels of subjective socioeconomic status, although social isolation was a positive predictor of BADL (*β*_simple_ = 4.738, *t* = 9.858, *p* < 0.001), its predictive effect was weaker. This suggests that social isolation is a diminishing predictor of BADL as the subjective socioeconomic status of the oldest old increases.

The predictive effect of social isolation on depression was not significant for the oldest old with low levels of subjective socioeconomic status (*β*_simple_ = −1.709, *t* = −1.794, *p* = 0.073), whereas the negative predictive effect of social isolation on depression was significant for the oldest old with high levels of SES (*β*_simple_ = −0.983, *t* = −2.045, *p* = 0.041), which suggests that subjective socioeconomic status has different moderating effects on social isolation and depression at different levels. In addition, as shown in Table 6, the mediating effects of BADL and depression between social isolation and cognitive ability increased with decreasing subjective socioeconomic status. In other words, the worse the subjective socioeconomic status of the oldest old, the more likely social isolation is to negatively impact their cognitive ability through BADL and depression.

**Table 6:** Mediating effects at different levels of SES.

Pathways	SES	Effect	Boot SE	Bootstrap 95% CI	
				Lower	Upper
SI → BADL→CA	Low	0.198	0.071	0.071	0.356
	High	0.098	0.035	0.035	0.172
SI → Depression → CA	Low	0.155	0.063	0.045	0.295
	High	0.041	0.017	0.011	0.078
SI → BADL→Depression→CA	Low	0.110	0.040	0.036	0.194
	High	0.055	0.019	0.017	0.094

*N* = 453.SES, subjective socioeconomic status; SI, social isolation; BADL, basic activities of daily living; CA, cognitive ability.

## 5 Discussion

To our knowledge, this is the first study to specifically target social isolation and cognitive ability among the oldest old in nursing homes in China during the post-COVID-19 era. This study not only discussed the prevalence of social isolation in this population but also constructed a moderated chain-mediated model to verify how BADL and depression act as chain mediators between social isolation and cognitive ability and how subjective socioeconomic status moderated this process. The results of the study showed that although the COVID-19 pandemic seems to be over, the prevalence of social isolation among the elderly in nursing homes remains high, up to 56.1%. In particular, isolation from friends (63.1%) is much higher than isolation from family (41.1%). Further analysis of mediating and moderating effects indicated that BADL and depression mediated the effects of social isolation on cognitive ability, while subjective socioeconomic status moderated the indirect pathway between social isolation and BADL and depression, respectively.

The present study found that the level of social isolation among the oldest old in nursing homes from China was significantly higher than that found in a large survey among the oldest old in a community (34.6%) from Germany (Moormann et al., 2024) and slightly higher than the results of a large survey of community-dwelling oldest old in China (54.0%) (Liu et al., 2022). These studies have shown that the oldest old in nursing homes suffer more significant social isolation than younger older adults in the same community (Wu and Sheng, 2020). This discrepancy stems from the fact that the oldest old themselves are inherently more vulnerable among older adults, facing higher rates of widowhood and poorer health status (American Psychological Association, 2021). In addition, it is also inevitably influenced by factors in the residential environment, such as changes in visit policies for nursing homes during and after epidemics (Barnett and Grabowski, 2020). Institutions mainly provide care services and pay less attention to the emotional needs of older people. Therefore, older people may struggle to establish close relationships with other members of the same institution, including caregivers and neighbors, to maintain appropriate social interactions. In addition, participants were generally more isolated from friends than from family, a finding that was consistent with previous research (Moormann et al., 2024). This phenomenon may be partly explained by the traditional Chinese family culture, where family members continue to visit older people regularly and provide necessary financial and emotional support even when they are admitted to nursing homes.

In contrast, maintaining or developing friendships is more difficult in institutions, not only because of changes in the health of old friends or social distance barriers but also because of the scarcity of new friends. Some studies (Chen et al., 2023) have shown that although the institutions provide environments where peers live together, institutionalized older people’s social networks do not expand. Instead, they feel more isolated than community-dwelling older adults. These findings suggest that when designing and implementing public health policies or research for older adults, more attention should be paid to enhancing the social support systems of older adults, particularly the construction and maintenance of friendship networks, especially for those individuals living in institutionalized settings.

Our study found that social isolation was strongly correlated with their subjective socioeconomic status, indicating that the worse the subjective socioeconomic status of the oldest old, the greater the social isolation they experience. This phenomenon is not only related to the availability of social resources due to socioeconomic status but may also be related to the perpetuation of economic inequalities exacerbated by the COVID-19 pandemic. This economic inequality not only results in increased objectification of social interactions but also widens psychological distance, lowering trust and further increasing the vigilance of social interactions between individuals (Cheng et al., 2024). Thus, social isolation is more prevalent in the economically disadvantaged elderly population, which may be due to the low value of interactions perceived by others. Furthermore, individuals with poor subjective socioeconomic status often experience greater economic pressure (Xie and Lv, 2023). Based on need theory and conservation of resources theory, in the face of such pressure, elderly individuals may prioritize their limited resources for basic needs such as medical care and food, while selectively reducing social activities to save economic resources. This reduction in social activities can lead to decreased interactions with the outside world, thereby increasing the risk of social isolation and subsequently elevating the likelihood of depression. Therefore, strengthening social support and increasing opportunities for interpersonal interaction among the oldest old is necessary to mitigate the negative effects of social isolation.

The present study not only found that social isolation positively predicted cognitive ability but also that it indirectly affected cognitive ability through BADL and depression. Nearly all hypotheses proposed in the present study were supported. In particular, the indirect effects acted primarily through the chain mediation of BADL and depression, as well as the independent mediation of BADL. In the post-COVID-19 era, the oldest old living in nursing homes continue to face severe challenges of social isolation, and prolonged social isolation severely affects their BADL and depression, which in turn negatively impacts their cognitive ability. Depression as a mediator between social isolation and cognitive ability has been widely discussed and validated in previous studies (Evans et al., 2019; Guo et al., 2021b), aligning with the findings of the present study. The present study adds new evidence to the study of the relationship between social isolation and cognitive ability by expanding the age group studied to include the oldest old in Chinese nursing homes during the post-COVID-19 era. In turn, these findings suggest new guidelines for public health and management practices in elderly care institutions. Given that prolonged social isolation may have a serious negative impact on cognitive ability, relevant policy-makers, and nursing home managers should recognize the importance of mental health support. For example, nursing homes can mitigate the adverse effects of social isolation on the cognitive ability of residents by organizing more social activities, providing mental and physical health support services, and training staff to recognize depression in older people and help them cope with it.

In addition, another factor that must be considered is BADL. Social isolation affects cognitive ability mostly through the independent effect of BADL and the chain-mediated effect of BADL and depression. Typically, older adults experiencing social isolation have a low quality or limited number of social relationships that are strongly associated with functional status, including somatic and cognitive ability (Ali et al., 2018). For example, some studies have assessed the functional status of older adults through objective metrics such as walking speed (Shankar et al., 2017) and grip strength (Yu et al., 2020) and found that those with more extensive social networks experienced less decline in these indicators. Instead, we used the Barthel index scale to assess whether the oldest old could perform certain activities to measure their functional status. The results were as expected: the oldest old with higher levels of social isolation had poorer physical functioning. This finding further supports the hypothesis that social relationships and somatic functional status are correlated in older adults and is consistent with previous research (Schrempft et al., 2019). Similarly, poor social relationships pose a threat to cognitive ability in older people (Luo et al., 2021), not only directly but also indirectly by affecting physical activity functioning, which is particularly evident in people aged 65 years and older (Hopper et al., 2024). A potential reason for this finding is that older people who are socially isolated may lack positive influences and supervision from family or friends, making them more likely to adopt unhealthy lifestyles (Watt et al., 2014), thereby exacerbating activity limitations (Sulander, 2011; Hotta et al., 2018). Once BADL is restricted, the rate of cognitive decline increases by 156%, along with an increased risk of depression (Rajan et al., 2013). Therefore, attempts to mitigate the impact of social isolation on cognitive ability in older adults must consider not only mental health issues but also the possibility of social isolation triggering a decline in somatic functioning and the subsequent negative consequences.

In the present study, we further hypothesized that subjective socioeconomic status may play moderating roles between social isolation and cognitive ability and between social isolation and BADL and depression. The results of the study confirmed that subjective socioeconomic status significantly moderated between social isolation and BADL and depression. This result was expected because the oldest old with lower socioeconomic status are more likely to be negatively affected by poor social relationships than those with higher status, which may be particularly reflected in their functional abilities. In other words, older adults with low socioeconomic status struggle to afford healthcare and nutrition-related expenses, leading to a heavier burden of disease and psychological distress. Older adults in low socioeconomic status often struggle to afford healthcare and nutritional expenses, leading to a greater burden of illness and psychological stress. This economic pressure also affects their ability to engage in and maintain rehabilitation exercises, as they may be unable to afford transportation costs or sustain the financial investment required for consistent exercise amidst economic difficulties (Yang et al., 2023). In addition, they are more likely to suffer from increased physical and psychological distress due to potentially inadequate social connections and support (Robert and House, 1996). One study noted that for older men, the risk of functional limitations is 5.36 times higher than normal when socioeconomic status and social participation are low (Nilsson et al., 2011).

However, the moderating role of subjective socioeconomic status between social isolation and cognitive ability was not supported in this study. Although multiple aspects of social relationships have been associated with cognitive decline (Piolatto et al., 2022), this relationship does not seem to differ among the oldest old living in nursing homes according to their subjective socioeconomic status. A related explanation may be that economic differences in community-dwelling older adults may amplify differences in social relational resources, with better-off older adults having more opportunities to participate in social activities and interact with others, thus helping to preserve their cognitive abilities (Deng and Liu, 2021). In contrast, we inferred that these differences would have less impact on social relational resources among the oldest old living in nursing homes and that they would face similar levels of social isolation regardless of their socioeconomic status. Although this finding needs to be considered with caution, future research should further test this point through larger samples and multi-center investigations.

### 5.1 Limitations

Some limitations of this study should be pointed out. First, this study relied on self-reports to obtain information about subjective socioeconomic status, which may introduce bias. Future research could rely on more precise and sophisticated assessment tools to obtain more accurate information about socioeconomic status. Second, the participants were from more economically developed regions of eastern China, and future research should be extended to other regions of China to increase the generalizability of the findings. Third, although we found social isolation can differ by gender, this study did not validate the hypothesized model in this population, given its tractability and interpretability, which needs to be further explored. Fourth, this study employed a cross-sectional design. Although this method can efficiently reveal the correlations between subjective economic status, social isolation, BADL, depression, and cognitive ability at a specific point in time, its inherent limitations cannot be overlooked. Specifically, the cross-sectional design fails to capture and reflect the fluctuations and changes of an individual’s subjective economic status over their entire lifespan and its profound impact on the aforementioned factors. In light of this, we suggest that future research should favor longitudinal designs to gain a deeper understanding of the complex causal relationships among these variables and their dynamic evolution over time.

## 6 Conclusion

This study reveals the higher levels of social isolation faced by the oldest old living in nursing homes in China during the post-COVID-19 era and the negative impact of social isolation on their cognitive ability. The findings highlight the further negative impact of social isolation on the cognitive ability of the oldest old by decreasing their BADL and exacerbating their levels of depression. This process is moderated by subjective socioeconomic status: the oldest old with lower subjective economic status were more likely to experience decreased cognitive ability through BADL and depression.

## Data Availability

The raw data supporting the conclusions of this article will be made available by the authors, without undue reservation.
